# An ecological approach to the development of an active aging measurement in urban areas (AAMU)

**DOI:** 10.1186/s12889-020-10036-5

**Published:** 2021-01-03

**Authors:** Azadeh Lak, Parichehr Rashidghalam, S. Nouroddin Amiri, Phyo K. Myint, Hamid R. Baradaran

**Affiliations:** 1 Faculty of Architecture and Urban Planning, Shahid Beheshti University, Tehran, 1983963113 Iran; 2 Faculty of Architecture and Urban Planning, Khalij-e- Fars University, Bushehr, Iran; 3 Ageing Clinical & Experimental Research Team, Institute of Applied Health Sciences, University of Aberdeen, Aberdeen, UK; 4 Department of Epidemiology, School of Public Health, Iran University of Medical Sciences, Tehran, Iran

**Keywords:** Active ageing measurement, Ecological approach, Delphi technique

## Abstract

**Background:**

An essential element in the process of “aging well” is the concept of Active Aging (AA). To propose an operational definition of Active Aging, the present study seeks to develop a new measurement tool through an ecological approach. The aim is to recognize significant indicators that play a role in assessing AA in urban areas.

**Methods:**

This study was conducted through a two-phase process of consensus-building: 1) identifying a set of indicators that were likely candidates for inclusion based on literature review, and 2) a two-round modified Delphi survey using an international panel of academic experts in environmental sciences and gerontology to achieve consensus on the importance of the extracted indicators and validate the items. The panelists were asked to complete a researcher-developed questionnaire with an 11-point Likert scale based on the indicators derived in phase 1. Finally, the Delphi survey’s valid indicators and criteria were utilized to develop the measurement tool.

**Results:**

At the outset, a list of 111 indicators of AA was prepared through the desk study. A panel of 22 experts reviewed the extracted items and arrived at a consensus on 99 items in the first round and finalised in the second round. Thematic analysis of the panelists’ open-ended responses revealed new concepts that would be explicitly considered by the consensus group. This developed measurement scale consists of five domains, i.e., individual, spatial, socio-economic, governance, and health-related, which contain 15 criteria and 99 indicators.

**Conclusions:**

The present researchers have developed the active aging measure for urban settlements (AAMU), which can be used both by policy-makers and as an informal self-reported statement among the elderly. AAM’s results in the elderly’s residential environmental communities can improve policy-making to address urban design to sustain an active, healthy life among older people in urban environments.

**Supplementary Information:**

The online version contains supplementary material available at 10.1186/s12889-020-10036-5.

## Background

The issue of long-term care has always been at stake in policy-making concerning the resources necessary to fulfil the care needs of the elderly. Due to the increased life expectancy delayed health decline due to medical advances, a gradual shift in care focus has occurred. In some countries, such as those in the European Union, there is a tendency to combine social behaviour and long-term care (referred to as ‘Active Aging’) to promote healthy aging [1, 2]. Active Aging (AA) can be defined as “the process of optimising opportunities for health, participation, and security to enhance the quality of life as people age” [3]. Central to this process is socially active engagement in different aspects of social, professional, and family life, including paid work, community activities, residential care, and leisure activities [4, 5] so that a harmonious relationship between life and activities could improve the health and well-being in old age [5, 6].

The idea of AA was initially proposed, emphasizing being active to maintain health and productivity [7]. Later on, it advocated for older adults’ right to make personal decisions, remain independent, and enhance their quality of life [3, 5]. Thus, although the elderly are not counted among the active labour force, they are considered contributors in their entire lives and encouraged to participate in various social and individual activities [8]. Recently, AA’s primary focus has shifted from physical health and ‘employability’ to engage with life in general [9].

In line with this, research has adopted multidimensional definitions of Active Aging and addressed various issues, including subjective and objective perception of health, affective and cognitive factors, functionality, and social status [10]. Multiple variables of participation, such as leisure [11], social engagement [10], and lifelong learning [12], have also been taken into account.

The concept of AA is now widely used in policy-making regarding the aging population and is measured employing the Active Aging Index (AAI). Accordingly, AAI has been used since 2012 by the European Union (EU) as a composite index, and its original purpose was ambiguous [13]. According to São José et al. (2017), AAI measures the level to which older people live independent lives, participate in paid employment and social activities, and their capacity to actively age. The index is constructed from 22 individual indicators grouped into four distinct domains in the 28 EU countries. Each domain presents a different aspect of measuring older people’s untapped potential for active and healthy ageing. They also argue that AAI, which is a narrowly conceptualised and under-theorised policy tool, is based on a restrictive model of Active Aging and serves the process of Model Aging. Furthermore, underlying AAI is a priori assumption concerning the capacity of older people in a European context as well as the activities and domains of life they tend to value. However, according to Foster and Walker (2015), we need to have new tools foster social inclusion, flexibility and respect for national and cultural diversity [14].

In reviewing the recent active aging assessment scales, We found a scale in Thailand containing the following domains: being self-reliant, being actively engaged with society, developing spiritual wisdom, building up financial security, maintaining a healthy lifestyle, engaging in active learning, and strengthening family ties to ensure care in later life [15]. This scale suggested culture-specific factors included to promote the elders’ well-being. Recently, the UJACAS questionnaire is claimed as a scale to evaluate active aging from the elders’ perspectives [16]. This questionnaire was developed based on four domains of active aging: the elders’ goals, the elders’ functional capacity, the elders’ autonomy, and the elders’ activities [16].

The present study seeks to develop a new measurement tool for AAI in urban areas. Against the background of emerging research interest in integrating Active Aging in long-term care policies by adopting an ecological approach to consider the community environments on active life among the elders. In this regard, it is aimed at providing an operational definition of AA through the Delphi method. As emphasised in the literature, the socio-cultural milieu is necessary to cross-culturally develop social policies and conceptual frameworks for aging populations [17] in the current AA measurement. Thus, a significant issue in the literature could be resolved, i.e., the rough treatment of the notion of AA. If used to predict activity level, AA measurement could help monitor the Active Aging status among older persons and evaluate the effectiveness of policy and service changes applied to encourage Active life in urban communities.

## Methods

According to Woodcock et al.(2020), a two-phase consensus-building approach was conducted: (1) identifying the list of features of measurement tools that were potential represents for inclusion based on importance (April–May 2019); (2) conduct of a modified two-round Delphi survey (June–August 2019). The Delphi method was chosen because it is one of the most suitable research approaches aimed at unknown subjects [18].

### Phase 1: identifying the candidate features to include in the measurement tool

**Fig. 1:**
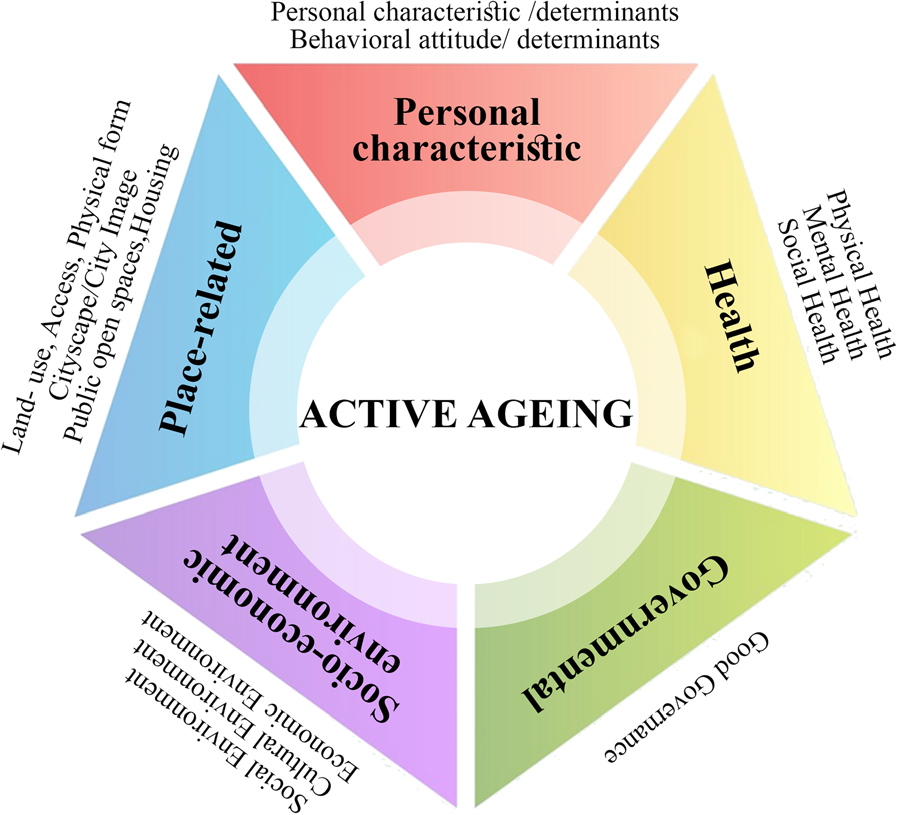
The Active aging framework

We extracted a list of criteria and indicators that could be proposed for the decisional operation of AA; all these indicators and criteria modified the Delphi study. We reviewed the existing literature in the development of AA measurement for improvement, including peer-reviewed and grey literature. We started with articles recommended by members of the steering group in Lak et al. (2020). The authors checked the reference list of these articles to identify other relevant studies in the next step. Finally, the different aspects of Active Aging which have been discussed in the literature can be categorised in the 5P Model: 1) person (personal status); 2) process (socio-economic environment); 3) place (built environment); 4) policy-making (governance); and Prime (health) [19]. This process resulted in a list of extracted indicators for inclusion in the modified Delphi study. For each indicator, we applied the supporting literature to draft an explanation of what the measurement tool might include (see Fig. 1, Table 1).

**Table 1:** The list of Dimensions, Criteria And Indicators with References from Desk study

Dimensions	Criteria	Indicators	References
Personal characteristic	Personal characteristic/determinants	age	[20–25]
		gender	
		Education	
		Ethnicity	
		Residential tenure	
		Marital status	
		Household size	
		Driving license	
		Employment/ paid work	
		Eating/drinking habitat	[25, 26]
		Family support/ domestic care	[25, 27]
		Self-care	[25, 28]
		Self-promotion	[25, 28]
		Mutual help/	[25, 28]
		Self-esteem	[25, 28]
		Life satisfaction	[25, 29]
		Travel behaviour	[25, 30]
	Behavioural attitude/ determinants	Smoking	[29]
		Alcohol	[25, 31]
		Length of activity	[25, 31]
Place-related	Land- use	Shopping services	[23, 25, 32–34]
		service proximity	[25, 35]
		public facility	[2, 23, 25]
		mix use	[23, 32, 34, 36–39]
		Facilities management	[40]
		sport recreation facility	[41, 42]
	Access	Connectivity	[3, 34, 43, 44]
		Accessibility services	[22, 23, 25, 38, 44–52]
		Traffic condition	
		Pavement condition	
		Walkable Environment	[3, 20, 23, 32–34, 38, 39, 41, 43–45, 49, 50, 52–66]
		Mobility	[3, 30, 42, 43, 52, 54, 60, 67–69]
		Transportation	[22, 23, 46, 51, 52, 57, 60, 64, 70]
	Physical form	Up keeping	
		Abandon buildings	
		Presence amenities/infrastructure sufficiency	
		Urban Block size	[34, 71, 72]
		Safety	[20, 22, 23, 32, 38, 49, 57, 64]
		Security	[22, 32, 33, 35, 43, 44, 46, 73, 74]
		green space	[3, 43, 44, 75]
	Cityscape/City Image	perceived distance	
		legibility	[53]
		Perceived Aesthetics	[3, 20, 23, 34, 36, 38, 49, 64, 73]
		Natural scenery	[23, 32]
	Public open spaces	Street lighting	[23, 43, 51, 76]
		Area open spaces ratio	[77]
		Recreation Public open spaces	[78]
		Quietness	
		cleanness	[3, 34, 44, 64, 73]
		maintenance	[44, 53]
		Pollution	[50]
		Landscaping quality	[3, 34, 43, 44, 51, 53]
	Housing	Universal design	[37, 57, 79, 80]
		Residential density	[32, 37–39, 81]
		Residential Care Facility	[59]
		Type of housing	[82–84]
Socio-economic environment	Social Environment	Life expectancy	[35]
		Quality life	[26, 42, 52, 59, 61, 81, 85]
		Social interaction/ community activities	[22, 44, 46, 86, 87]
		Happiness	[88]
		Social inclusion	[32, 35, 43, 46, 49, 71, 89]
		Social Inequalities	[58]
		Social Demography	[20, 90]
		Social democracy	[32, 91]
		Participation	[21, 22, 30, 33, 54, 67, 71, 74, 92–95]
		Social Support	[31, 41, 44, 49]
		Education learning	[31]
		Social capital	[49]
	Cultural Environment	Religious activity	[33, 43, 51, 67, 73, 75]
		Cultural events	
		Sense place	
	Economic Environment	healthcare services	[22, 31, 51, 74]
		limited income	[31]
		insurance coverage	[31]
		Socio-economic status	[20, 32]
		Affordable housing	[22]
		Car ownership	[32]
		Homeownership	[85]
		Household income	[25]
		Living situation	[25, 32, 59]
Governmental	Good Governance	Effective collaboration	[79, 96–102]
		Performance orientation	[47, 59, 62, 101–103]
		governance	
		Equity	
Health	Physical Health	Disability	[30, 42, 55]
		Public Health	[25, 30, 33, 43, 51, 56, 66, 76]
		Incidence of disease	[25]
		Pain feeling	[25]
		Functional ability	[25]
		Risk institutionalization	[25]
		Self-reported falls	[24, 25]
		Self-reported health	[25]
		Physical activity	[25, 33, 74, 81, 85]
		Activities daily living	[25]
		Genetic factors	[25]
		BMI	[25, 56]
		Sleep hygiene	[28]
		Personal hygiene	[28]
	Mental Health	Depressive	
		Cognitive functioning	[25, 30, 51, 54, 76, 81]
		Psychological distress	
		Psychological well-being	
		Anxiety	
		Anger	
		Restorative activity	
		Spiritual activity	
		Self-actualization	[43, 104]
	Social Health	Relation family	[30, 32, 59, 105, 106]
		Relation work	
		social life	
		sense community	[87, 105]

Our Delphi questionnaire was developed using the indicators extracted from the literature in the Desk study. Likert-scale items were designed to collect both qualitative and quantitative data, and some open-ended questions were developed to allow for further qualitative input. Certain aspects of the topic were investigated through open questions. Using the Likert scale is a suitable tool for measuring the indicators and criteria in questions, and it helps to evaluate the level of consensus quickly. We used the 11-item Likert scale in this study, as Cronbach alpha coefficient is highest for 11-point Likert scale and superior to others [107].

**Fig. 2:**
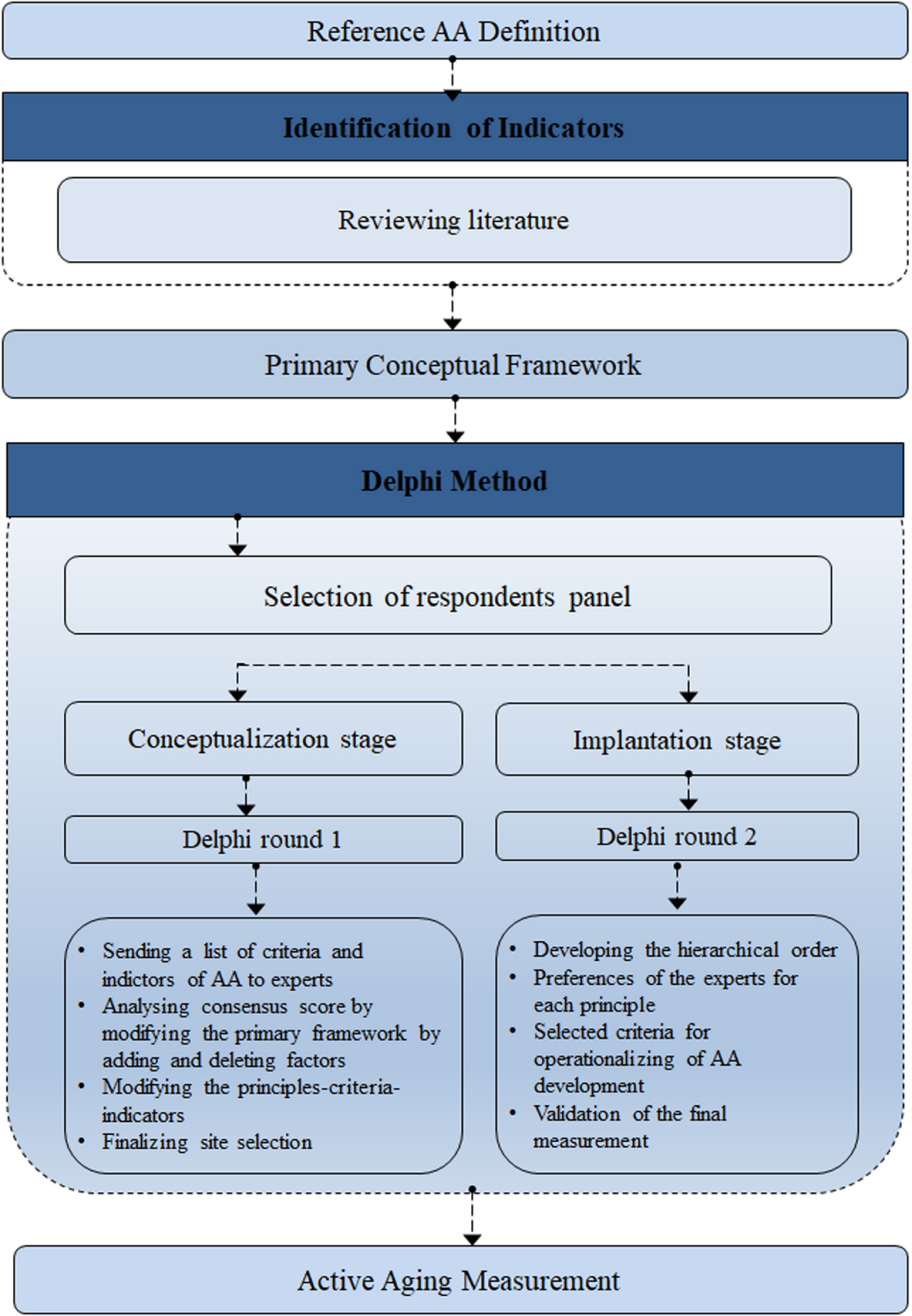
The Research Process

First, a pilot study was conducted using four experts to examine the questions’ comprehensibility and its usability. Also, the design of the study was validated by four experts as gerontologists and urban designers. The feedback from the pilot study was taken into account in the final version of the questionnaire. Some of the criteria were eliminated in this phase to reduce the final questionnaire items down to 111. This process is shown in Fig. 2.

### Phase 2: consensus-building study to select and refine the indicators and criteria

The Delphi technique [108] was applied to build consensus on which indicators from phase1 1 were significant to include as features of a AA measurement tool, using two rounds of scoring and review by an expert panel over eight weeks.

The feedback process is integrated into the Delphi method. The first round results are revisited and possibly modified by the respondents in later phases when they have reviewed and considered the feedback of the other Delphi experts [109]. The characteristic of this method is that it ensures the anonymity of respondents. The administrator controls the feedback process, and there are several statistical techniques for interpreting the data [110]. As the Delphi procedure progresses, the respondents tend to present their opinions with more in-depth insight. Research has shown that a rational number of rounds, or iterations, to reach a consensus is usually two or three [18]. The process can be followed in Fig. 2.

A recent study has suggested that a panel of 10–18 experts is enough for a homogeneous sub-group [111]. However, if a Delphi study involves a range of reference groups, a more significant number of experts is usually needed. Qualitative research in social sciences usually requires 15–30 experts [112], while it is quite common for other types of research to use 15–20 respondents [18, 113].

For a heterogeneous sample, a group of 20–50 participants seems large enough to ensure variety in opinions and small enough to ensure consensus [114]. To achieve this number, we communicated with 45 experts around the world, and, finally, 22 experts agreed to participate in two rounds of research. In doing so, we sent the invitation email to the 45 experts we had identified along with the necessary information about the survey topic. A reminder was sent out two weeks after the initial request emails. The process was repeated three times in two-week intervals, and, after the eighth week, 22 experts completed the survey in two rounds. As our expectation of the number of experts was met, the first round was finished. Next, an email was sent to the 22 experts who had been selected for round 1 to invite them to the second round. After three reminder emails, 22 experts answered the questions in round 2. This response rate was acceptable regarding the Delphi studies in the literature. The experts had two weeks in each round to complete the questionnaire [115].

#### Round 1

The essential Survey tool was sent to the 45 experts as an online questionnaire; however, 22 people agreed to participate in this study in two or three rounds. Given the variety in the participants’ backgrounds, they were informed about the project, research aims, several keywords, and the general framework. They were asked to score the importance of the indicators on an 11-item Likert scale (Additional file 1). Taking the SD below two more efficiently needs to define the 11-points Likert-scale questionnaire [114].

A total of 111 indicators and 15 criteria were derived from the desk study. The criteria were introduced as*:“ 1) personal characteristics, 2) behavioural attitude, 3) land use, 4) access, 5) physical form, 6) cityscape/city image, 7) public open spaces, 8) housing, 9) social environment, 10) cultural Environment, 11) economic environment, 12) good governance, 13) physical health, 14) mental health, and 15) social health. The ecological themes of Active Aging can be represented as the 5P model, which consists of person, processes, place, prime, and policy-making”* [1].

The experts also added several indicators to our list through their answers to open-ended questions. These new items were included in the questionnaire of round 2.

#### Round 2

Round 2 was conducted among those panellists who had participated in round 1. The structure of the questionnaire resembled that of round 1. The modified framework was presented to the participants along with a statistical summary (i.e., mean values) of the results of round 1. Besides, the questionnaires were customised for the participants to access their previous answers and the experts’ items. Finally, the questionnaire of round 2, which consisted of 100 items, was administered to the experts. This feedback process was carried out to encourage the panellists to reconsider the ratings to reach a consensus.

#### Data analysis

The results of the questionnaires of both rounds were keyed in and analysed using the Statistical Package for Social Sciences (SPSS) version 22.0 for Windows. We mainly focused on the descriptive analysis of the data, particularly central tendency (means) and level of dispersion (SD). The 11 anchors of the Likert scale ranging from 1: “Not at all Important” or “Not at all suitable” to 11: “Extremely Important” or “Extremely suitable” were used to determine the ‘importance’ and ‘suitability’ of each attribute. Importance indicates how much the experts regarded the feature in question as essential for AA’s assessment. Open-ended questions were included in line with Taylor and Judd’s recommendation (1989) to collect additional information to clarify the problems at hand. The experts were also asked to make suggestions to enhance the framework and add further criteria and indicators not listed in the questionnaire. Based on the modifications resulting from the comments received in round 1, the participants repeated their assessment in round 2. As the two rounds proved sufficient for achieving a consensus that was confirmed by statistical analysis, further rounds were not needed.

## Results

### Delphi panelists and participants response

Due to the interdisciplinary nature of Active Aging, we tried to determine the participants’ profiles by using a heterogeneous selection of experts. The 22 participants in this study were experts in urban design (23%), architecture (13%), urban planning (13%), landscape architecture (13%), gerontology (19%), and geriatrics (19%).

The participants were already familiar with different aspects of Active Aging and AAM from an ecological point of view. The participants were selected from locations as geographically diverse as Asia, Europe, America, and Australia to incorporate heterogeneous opinions into the new Active Aging measurement. This variety had a considerable effect on selecting indicators and enriched the new assessment tool due to the opinions’ critical nature.

Moreover, Cronbach’s α was measured to examine the reliability of the self-developed questionnaire (Supplementary File 1) and the internal consistency of opinion among the Delphi panellists. “To ensure the reliability of the data for further analysis, the study initially measured the validity of the Cronbach’s α and SD to measure the level of agreement in each round” [6, 110]. The results showed that the Cronbach’s alpha was 0.71 in round 1 and 0.79 in round 2.

Open-ended questions in the first stage of Delphi with the addition and subtraction of some concepts are considered valid measurement tools, mostly since 22 people answered positively to the extracted indicators in two rounds of the Delphi technique.

### The Delphi survey results

#### Round 1

In round 1, the experts scored the 15 criteria and 111 indicators in terms of five dimensions. Furthermore, 20 additional criteria were also suggested through the open-ended items in the questionnaire. The majority of the suggestions included modifying the definitions or labels of the existing indicators, proposing new indicators, and re-arranging the framework’s indicators. For example, the term ‘paid work’ was suggested instead of ‘limited work’ and ‘life scheme’ instead of ‘self-esteem.’ The indicators that were added according to the suggestions include ICT use, art activities, having a pension, household expenditure, comfort, lifelong learning, leisure activities and recreational activities, living at least with one child, and functionality/indecency in activities.

Some other indicators were also suggested in different classifications. After the fundamental quantitative analysis, the reliability of the questionnaire was measured using Cronbach’s alpha. The value was 0.98, which is higher than the minimum threshold of 0.7 and indicates high internal consistency and reliability.

In our quantitative analysis, we measured SD, mean values, and general agreement. The results are summarised in Table 2. Based on the results of round 1, the indicators with an SD greater than 2.5 and a mean less than six were eliminated. The indicators removed in this phase include ethnicity, residential tenure, household size, driving license, self-promotion, smoking, alcohol, facility management, social demography, social democracy, insurance coverage, homeownership, household income, living situation, performance orientation, risk institutionalisation, and genetic factors.

**Table 2:** Descriptive Statistics (Round 1)

Dimensions	Criteria	Indicators	N	Minimum	Maximum	Mean	Std. Deviation	Rate of Agreement
Personal characteristic	Personal characteristic /determinants	age	23	0	11	8.00	2.000	0.727
		gender	23	0	11	7.00	2.000	0.636
		Education	23	0	11	6.00	2.000	0.545
		Ethnicity	23	0	11	5.05	3.000	0.459
		Residential tenure	23	0	10	5.00	3.000	0.455
		Marital status	23	0	11	7.00	2.500	0.636
		Household size	23	0	11	5.00	3.000	0.455
		Driving license	23	0	11	6.00	3.000	0.545
		Employment/ paid work	23	0	11	7.00	2.500	0.636
		Eating/drinking habitat	23	1	11	7.00	2.500	0.636
		Family support/ domestic care	23	5	11	9.00	1.000	0.818
		Self-care	23	0	11	7.00	2.500	0.636
		Self-promotion	23	0	11	6.00	3.000	0.545
		Mutual help/	23	0	11	6.00	2.000	0.545
		Self esteem	23	0	11	7.00	2.000	0.636
		Life satisfaction	23	0	11	7.00	2.000	0.636
		Travel behavior	23	0	11	7.00	2.000	0.636
	Behavioural attitude/ determinants	Smoking	23	0	11	6.00	3.000	0.545
		Alcohol	23	0	11	6.00	3.000	0.545
		Length of activity	23	5	11	9.00	1.000	0.818
Place-related	Land- use	Shopping services	23	0	11	7.00	2.000	0.636
		service proximity	23	0	11	7.00	2.000	0.636
		public facility	23	1	11	7.00	2.000	0.636
		mix use	23	0	11	6.00	2.500	0.545
		Facilities management	23	0	11	5.00	3.000	0.455
		sport recreation facility	23	5	11	8.00	2.500	0.727
	Access	Connectivity	23	0	11	8.09	2.000	0.735
		Accessibility services	23	0	11	7.00	2.000	0.636
		Traffic condition	23	0	11	7.00	2.000	0.636
		Pavement condition	23	2	11	8.09	2.000	0.735
		Walkable Environment	23	1	11	8.00	2.000	0.727
		Mobility	23	0	11	7.00	2.500	0.636
		Transportation	23	1	11	7.00	2.000	0.636
	Physical form	Up keeping	23	5	11	7.00	1.000	0.636
		Abandon buildings	23	0	11	7.00	2.000	0.636
		Presence amenities/infrastructure sufficiency	23	5	11	8.00	1.000	0.727
		Urban Block size	23	0	11	7.00	2.000	0.636
		Safety	23	0	11	7.00	2.000	0.636
		Security	23	2	11	8.00	2.000	0.727
		green space	23	3	11	8.00	2.000	0.727
	Cityscape/City Image	perceived distance	23	4	11	8.00	2.000	0.727
		legibility	23	0	11	7.00	2.000	0.636
		Perceived Aesthetics	23	2	10	7.00	2.000	0.636
		Natural scenery	23	2	11	8.00	2.000	0.727
	Public open spaces	Street lighting	23	0	11	7.00	2.000	0.636
		Area open spaces ratio	23	0	11	7.05	2.000	0.641
		Recreation Public open spaces	23	2	11	7.00	2.000	0.636
		Quietness	23	0	11	7.00	2.000	0.636
		cleanness	23	2	11	8.00	2.000	0.727
		maintenance	23	1	11	7.00	2.000	0.636
		Pollution	23	1	11	8.09	2.000	0.735
		Landscaping quality	23	1	11	7.00	2.000	0.636
	Housing	Universal design	23	0	11	6.00	2.500	0.545
		Residential density	23	0	11	6.00	2.500	0.545
		Residential Care Facility	23	0	11	7.00	2.500	0.636
		Type of housing	23	0	11	7.00	2.000	0.636
Socio-economic environment	Social Environment	Life expectancy	23	0	11	7.00	2.500	0.636
		Quality life	23	0	11	8.00	2.500	0.727
		Social interaction/ community activities	23	3	11	9.09	2.000	0.826
		Happiness	23	1	11	7.00	2.000	0.636
		Social inclusion	23	1	11	8.00	2.000	0.727
		Social Inequalities	23	2	11	6.00	2.000	0.545
		Social Demography	23	0	11	6.00	3.000	0.545
		Social democracy	23	0	11	7.05	3.000	0.641
		Participation	23	2	11	7.00	2.000	0.636
		Social Support	23	2	11	8.00	2.000	0.727
		Education learning	23	2	11	7.00	2.000	0.636
		Social capital	23	1	11	7.00	2.000	0.636
	Cultural Environment	Religious activity	23	0	11	8.05	2.000	0.732
		Cultural events	23	0	11	7.00	2.000	0.636
		Sense place	23	1	11	7.00	2.500	0.636
	Economic Environment	healthcare services	23	1	11	8.05	2.000	0.732
		limited income	23	0	11	7.00	2.500	0.636
		insurance coverage	23	0	11	7.00	3.000	0.636
		Socioeconomic statues	23	0	11	7.00	2.500	0.636
		Affordable housing	23	1	11	7.00	2.000	0.636
		Car ownership	23	0	11	6.00	2.000	0.545
		Homeownership	23	0	11	7.00	3.000	0.636
		Household income	23	0	11	6.00	3.000	0.545
		Living situation	23	0	11	6.00	3.013	0.545
Governmental	Good Governance	Effective collaboration	23	2	11	8.00	2.087	0.727
		Performance orientation	23	0	11	6.00	3.000	0.545
		governance	23	0	11	6.00	2.500	0.545
		Equity	23	0	11	7.00	2.500	0.636
Health	Physical Health	Disability	23	0	11	8.00	2.000	0.727
		Public Health	23	0	11	8.00	2.500	0.727
		Incidence disease	23	0	11	8.00	2.000	0.727
		Pain feeling	23	2	11	8.00	2.000	0.727
		Functional ability	23	0	11	8.00	2.000	0.727
		Risk institutionalization	23	0	11	6.00	3.000	0.545
		Self-reported falls	23	2	11	7.00	2.000	0.636
		Self-reported health	23	2	11	7.00	2.000	0.636
		Physical activity	23	0	11	7.00	2.500	0.636
		Activities daily living	23	0	11	8.00	2.000	0.727
		Genetic factors	23	1	11	7.00	3.000	0.636
		BMI	23	1	11	7.00	2.000	0.636
		Sleep hygiene	23	1	11	7.00	2.000	0.636
		Personal hygiene	23	1	11	6.00	2.000	0.545
	Mental Health	Depressive	23	1	11	8.05	2.000	0.732
		Cognitive functioning	23	7	11	9.09	1.000	0.826
		Psychological distress	23	1	11	8.00	2.000	0.727
		Psychological wellbeing	23	3	11	8.00	2.000	0.727
		Anxiety	23	0	11	7.00	2.500	0.636
		Anger	23	1	11	7.00	2.000	0.636
		Restorative activity	23	1	11	8.00	2.000	0.727
		Spiritual activity	23	0	11	7.00	2.000	0.636
		Self-actualization	23	0	11	7.00	2.000	0.636
	Social Health	Relation family	23	0	11	8.00	2.500	0.727
		Relation work	23	0	11	8.00	2.500	0.727
		social life	23	1	11	8.00	2.000	0.727
		sense community	23	1	11	8.00	2.000	0.727

Finally, the items were reduced from 111 to 99 by eliminating 18 items (yellow line in Table 1), and ten new indicators (Green line in Table 2) were added to the questionnaire based on our qualitative analysis. The final questionnaire in round 2 contained 100 indicators and 15 criteria. Also, the questionnaire was slightly modified following the suggestions of some experts. For instance, self-esteem was moved to the dimension of psychological health.

#### Round 2

Table 3 presents the descriptive statistics (mean and SD) for the questionnaire completed by 22 experts in the second round. The results are indicative of a 51% reduction in SD in round 2 of the Delphi survey. The indicators’ mean value has increased compared to the first round, and there is a significant difference in specific agreement between the two rounds. Besides, Kendall’s w indicates that the overall agreement’s mean value has increased from 0.75 to 0.81 in the second round. It should be noted that Kendall’s w lies in a range from 0 (no agreement) to 1 (complete agreement). The authors decided to finish the Delphi survey after the second round because the difference between the two rounds was less than 20%, confirming a stable situation. Furthermore, most indicators reached a high level of a specific agreement, which is indicated by the value of Kendall’s w that shows the difference between the means in the two rounds of the Delphi survey.

**Table 3:** Descriptive Statistics (Round 2)

Dimensions	Criteria	Indicators	N	Minimum	Maximum	Mean	Std. Deviation	Rate of Agreement
Personal characteristic	Personal characteristic /determinants	age	23	0	11	8.50	2.00	0.773
		gender	23	0	11	8.00	2.00	0.727
		Education	23	0	11	6.50	2.00	0.591
		Marital status	23	0	11	7.00	2.00	0.636
		Employment/ paid work	23	0	10	7.50	2.00	0.682
		Eating/drinking habitat	23	0	11	8.00	2.00	0.727
		Family support/ domestic care	23	0	11	9.00	1.00	0.818
		Self-care	23	0	11	8.00	2.00	0.727
		Mutual help/ Having a Partner	23	0	11	8.00	2.00	0.727
		Life satisfaction	23	1	11	7.50	2.00	0.682
		Travel behavior	23	5	11	7.50	2.00	0.682
		living at least with one child	23	0	11	6.00	2.00	0.545
	Behavioural attitude/ determinants	Functionality/ indecency in activities	23	0	11	6.00	2.00	0.545
		Length of activity	23	0	11	9.00	1.00	0.818
Place-related	Land- use	Shopping services	23	0	11	7.50	2.00	0.682
		service proximity	23	0	11	7.00	2.00	0.636
		public facility	23	0	11	7.00	2.00	0.636
		mix use	23	0	11	8.00	2.00	0.727
		sport recreation facility	23	0	11	8.50	2.00	0.773
	Access	Connectivity	23	0	11	8.09	2.00	0.735
		Accessibility services	23	0	11	7.50	2.00	0.682
		Traffic condition	23	5	11	7.50	2.00	0.682
		Pavement condition	23	0	11	8.09	2.00	0.735
		Walkable Environment	23	0	11	8.00	2.00	0.727
		Mobility	23	1	11	7.50	2.00	0.682
		Transportation	23	0	11	8.00	2.00	0.727
	Physical form	Up keeping	23	0	11	8.00	1.00	0.727
		Abandon buildings	23	5	11	7.00	2.00	0.636
		Presence amenities/ Infrastructure sufficiency	23	0	11	8.00	1.00	0.727
		Urban Block size	23	0	11	7.50	2.00	0.682
		Safety	23	0	11	7.50	2.00	0.682
		Security	23	2	11	8.00	2.00	0.727
		green space	23	1	11	8.00	2.00	0.727
	Cityscape/City Image	perceived distance	23	0	11	8.00	2.00	0.727
		legibility	23	1	11	8.00	2.00	0.727
		Perceived Aesthetics	23	5	11	7.00	2.00	0.636
		Natural scenery	23	0	11	8.00	2.00	0.727
	Public open spaces	Street lighting	23	5	11	7.00	2.00	0.636
		Area open spaces ratio	23	0	11	8.00	2.00	0.727
		Recreation Public open spaces	23	0	11	7.00	2.00	0.636
		Quietness	23	2	11	7.50	2.00	0.682
		cleanness	23	3	11	8.00	2.00	0.727
		maintenance	23	4	11	7.50	2.00	0.682
		Pollution	23	0	11	8.09	2.00	0.735
		Landscaping quality	23	2	10	7.00	2.00	0.636
	Housing	Universal design	23	2	11	6.00	2.50	0.545
		Residential density	23	0	11	6.00	2.00	0.545
		Residential Care Facility	23	0	11	8.00	2.00	0.727
		Type of housing	23	2	11	7.00	2.00	0.636
Socio-economic environment	Social Environment	Life expectancy	23	0	11	7.00	2.00	0.636
		Quality life	23	2	11	8.00	2.00	0.727
		Social interaction/ community activities	23	1	11	9.09	2.00	0.826
		Happiness	23	1	11	7.50	2.00	0.682
		Social inclusion	23	1	11	8.00	2.00	0.727
		Social Inequalities	23	0	11	6.00	2.00	0.545
		Social Participation	23	0	11	7.00	2.00	0.636
		Social Support	23	0	11	8.00	2.00	0.727
		lifelong learning	23	0	11	7.00	2.00	0.636
		Social capital	23	0	11	7.50	2.00	0.682
		Religious activity	23	0	11	8.05	2.00	0.732
		Cultural events/ activities	23	3	11	7.50	2.00	0.682
		Sense of place	23	1	11	7.00	2.00	0.636
	Economic Environment	health care services	23	1	11	8.05	2.00	0.732
		limited income/ paid work	23	2	11	7.00	2.00	0.636
		Having pension	23	0	11	7.00	2.00	0.636
		Household expenditure	23	0	11	7.50	2.00	0.682
		ICT use	23	2	11	7.00	2.00	0.636
		Affordable housing	23	2	11	7.50	2.00	0.682
Governmental	Good Governance	Effective collaboration	23	2	11	8.00	2.00	0.727
		Good governance	23	1	11	7.00	2.00	0.636
		Equity	23	0	11	7.50	2.00	0.682
Health	Physical Health	Disability. At least one ADL Disability	23	0	11	8.00	2.00	0.727
		Public Health	23	2	11	8.50	2.00	0.773
		Incidence disease	23	2	11	8.00	2.00	0.727
		Pain feeling	23	1	11	8.50	2.00	0.773
		Functional-ability	23	1	11	8.00	2.00	0.727
		Self-reported falls	23	0	11	8.00	2.00	0.727
		Self-reported health	23	0	11	7.50	2.00	0.682
		Art activities	23	0	11	7.00	2.00	0.636
		Leisure activities. Recreational activities	23	0	11	7.00	2.00	0.636
		Physical activity	23	1	11	7.00	2.00	0.636
		Activities daily living Level (ADL)	23	0	11	8.00	2.00	0.727
		BMI	23	0	11	7.50	2.00	0.682
		Sleep hygiene	23	0	11	7.00	2.00	0.636
		Personal hygiene	23	0	11	7.00	2.00	0.636
	Mental Health	Depression	23	2	11	8.05	2.00	0.732
		Cognitive functioning	23	0	11	9.00	1.00	0.818
		Psychological distress	23	0	11	8.00	2.00	0.727
		Psychological wellbeing	23	0	11	8.00	2.00	0.727
		Anxiety	23	0	11	7.00	2.00	0.636
		Anger	23	0	11	7.50	2.00	0.682
		Restorative activity	23	0	11	8.00	2.00	0.727
		Spiritual activity	23	2	11	7.50	2.00	0.682
		Self-esteem. Life scheme	23	0	11	7.50	2.00	0.682
		Self-efficacy	23	0	11	7.00	2.00	0.636
	Social Health	Relation in family	23	2	11	8.00	2.00	0.727
		Relation in work	23	2	11	8.00	2.00	0.727
		social life	23	0	11	8.00	2.00	0.727
		sense of community	23	0	11	8.00	2.00	0.727

## Discussion

We have developed an innovative measurement to imply the values of active aging characteristics based on the ecological model in both research or practice, as well as a self-rated and expert-based tool. Accordingly, the Active Aging Measurement in Urban Areas (AAMU) indicates the critical information provided by taking different levels of the environment, personal circumstances, and their relations into account in the elder’s life and culture-specific approach. The substantial difference between this tool and previous studies arises from the understanding that before we can evaluate the individual aspect of active aging, we need to define the nature of active aging in the relationship between person and environment. This measurement can assess active aging at the individual, spatial, socio-economic, governance, and the elders’ health levels. Also, this study was conducted the Delphi technique by applying 22 academic experts’ views with different knowledge in active aging to emerge the consensus based on a multidisciplinary approach in urban areas. Applying the context-based approach to understand the elders’ needs and preferences and considering the experts’ point of views to meet the elderly need to promote active aging, could help to develop the holistic measures.

Our findings represent Active Aging as a notion that begins at an individual layer (person) and involves personal characteristics and behavioural attitudes, segments of the social or physical environment, and a policy-making environment that differentiated this novelty from other studies. These factors are closely interrelated and need to function in tandem to fulfil Active Aging in a particular social, cultural, and religious system [19].

Such conception builds upon an ecological model [2, 116, 117] that focuses on the relations among the environmental levels of an aging person in the five dimensions while considering the micro (person), meso (process), and macro systems (place and policy-making) in terms of the health dimension), along with the vulnerable balance between individual competence and the environment in old age [19]. The nexus of cultural, social, and economic factors play a significant role in Active Aging and has a strong effect on social, incredibly emotional relationships in old age [118].

In this study, we indicated that lifelong learning is another essential factor influencing the elderly’s well-being. Given that productive activities could be conceived as a kind of social participation, merely caring for older people did not prove to be an adequate solution in this model. A possible explanation is that long-term care of older people who are ill or dependent may negatively affect the caregivers’ psychological well-being [119] or physical and mental health [119].

Furthermore, life satisfaction, which depends upon an individual’s cognitive evaluation of one’s life, may affect policy-making strategies. This satisfaction is influenced by an older adult’s perception of the quality of life and his or her private experiences [118]. Moreover, the way older people obtain life satisfaction may also be quite different from younger adults, e.g., by preferring emotionally close relationships to other social activities [120]. Nevertheless, lower life satisfaction has been found in specific cases, such as reduced self-care capacity or older caregivers. The results are affected by self-care capacity, the level of self-reported health, and dissatisfaction with social relationships, which are all based on the older adult’s perceptions [118]. Besides, the significance of ICT has also been indicated as a relatively new variable that forms active aging [119]. Currently, ICT is widely regarded as a predictor variable for Active Aging, improving older people’s well-being and increasing their engagement with life [119].

The cultural and social notion of Active Aging, with its contextual nature [121], has arisen out of environmental contexts’ diversity to clarify how a person reacts to and interacts with their environment [117, 122]. As suggested by the findings of our study, Active Aging results from personal, socio-cultural, and governance environments that are directly associated with the indicators of individual, environmental, social, economic, institutional, health-related factors.

Besides, this study identified a set of indicators for assessing those characteristics of quality of place that might contribute to Active Aging. These include measures of land use characteristics (deprivation or poverty in an area and neighbourhood degradation), physical form (neighbourhood degradation, accessibility to services and facilities, accessibility of public green space, walkability or pedestrian-friendliness, and housing type), security against perceived crime and anti-social behaviour, traffic safety, quality of public spaces, aesthetic aspects of architecture, landscape (lighting and furniture), pedestrian-friendly features, availability of sitting facilities (e.g., benches) and restrooms (toilets in public open spaces), hazards for pedestrians(sidewalks), home and environmental adaptations, climatic comfort, topography, and other desirable physical attributes like trees and green areas which promote a sense of support resilience and well-being [19].

### Limitations

This study faced the following limitations. The limitation is related to the reliability and validity of how expertise area categories were determined to select and invite the expert group. While statistical analyses and the literature generally support our findings, different experts from other areas might have provided different inputs to alter the study results. There were also inherent limitations in this study. 1) The findings from our Delphi survey must be validated by cross-sectional studies to verify the causal relationships among the variables. Of course, our assessment tool builds upon the knowledge provided by a wide range of agents (i.e., policy-makers, researchers, and the elderly). It may be an acceptable representation of the various elements of Active Aging discussed in the literature. 2) As the data concerning most of the variables were collected and assessed by self-reporting, subjective perceptions might have affected the results. Moreover, our sample was culturally homogeneous; therefore, further studies are required to validate the developed assessment tool in various cultural contexts.

Due to the different environments in urban and rural settlements, these environments have varied in economic, institutional, and sociodemographic structures in local Communities to create different lifestyles, needs, and expectations in the elderly. Therefore, to meet the needs and promote active aging, the developed measurement might be dissimilar in the elders’ lives, especially in the live hood and living costs. SO, this study is limited to develop a measurement tool for urban communities.

### Strengths

As discussed, the elderly’s capabilities. Thus, our proposed instrument seeks to offer clear guidance to policy-makers, which conforms well to older adults, experts, and local authorities’ opinions. The characteristic of this study is the most practical nature and real-world implications.

An expert-based and self-rated assessment tool was developed to measure Active Aging on an individual/environmental scale (micro, meso, and macro-level). Another contribution is the conception of Active Aging, which has its roots in the literature (through the notion of healthy and productive Aging) and involves a remarkably more extensive range of activities. Besides, it regards people with disabilities as active agers [123]. According to our findings, which are based on the ideas of policy-makers, researchers, and the elderly, Active Aging can be measured at five levels. We can argue that Active Aging is a higher-order construct consisting of five categories: personal, place-based, socio-economic, governance, and health-related indicators.

## Conclusion

The present researchers have developed the active aging measure for urban settlements (AAMU), which was can be used both by policy-makers and as an informal self-reported statement among the elderly. AAM’s results in the elderly’s residential environmental communities can improve policy-making to address urban design to sustain an active, healthy life among older people in urban environments.

## Supplementary Information

**Additional file 1.** Questionnaire for Measuring Active Aging. The online Questionnaire in the first round of Delphi.

## Data Availability

The datasets used and/or analysed during the current study available from the corresponding author on reasonable request.

## Abbreviations

AAM: Active Aging Measurement
WHO: World Health Organization
AA: Active Aging
AAI: Active Aging Index
