# Supplementary material for: An ecological approach to the development of an active aging measurement in urban areas (AAMU)
Source: BMC Public Health. 2021 Jan 3;21:4. doi: 10.1186/s12889-020-10036-5 (PMC7778809; doi:10.1186/s12889-020-10036-5)
Supplement: Supplementary file 1 — Additional file 1. Questionnaire for Measuring Active Aging. The online Questionnaire in the first round of Delphi. [file 12889_2020_10036_MOESM1_ESM.docx]

**Additional File 1:**

Questionnaire for Measuring Active Aging

The online Questionnaire in the first round of Delphi

| **Dimensions** | **Criteria** | **Indicators** | Score the degree of importance (1 to 11) | | | | | | | | | | |
| --- | --- | --- | --- | --- | --- | --- | --- | --- | --- | --- | --- | --- | --- |
| Personal characteristic | Personal characteristic | age |  |  |  |  |  |  |  |  |  |  |  |
|  |  | gender |  |  |  |  |  |  |  |  |  |  |  |
|  | /determinants | Education |  |  |  |  |  |  |  |  |  |  |  |
|  |  | Ethnicity |  |  |  |  |  |  |  |  |  |  |  |
|  |  | Residential tenure |  |  |  |  |  |  |  |  |  |  |  |
|  |  | Marital status |  |  |  |  |  |  |  |  |  |  |  |
|  |  | Household size |  |  |  |  |  |  |  |  |  |  |  |
|  |  | Driving license |  |  |  |  |  |  |  |  |  |  |  |
|  |  | Employment/ paid work |  |  |  |  |  |  |  |  |  |  |  |
|  |  | Eating/drinking habitat |  |  |  |  |  |  |  |  |  |  |  |
|  |  | Family support/ domestic care |  |  |  |  |  |  |  |  |  |  |  |
|  |  | Self-care |  |  |  |  |  |  |  |  |  |  |  |
|  |  | Self-promotion |  |  |  |  |  |  |  |  |  |  |  |
|  |  | Mutual help/ |  |  |  |  |  |  |  |  |  |  |  |
|  |  | Self-esteem |  |  |  |  |  |  |  |  |  |  |  |
|  |  | Life satisfaction |  |  |  |  |  |  |  |  |  |  |  |
|  |  | Travel behaviour |  |  |  |  |  |  |  |  |  |  |  |
|  | Behavioural attitude/ determinants | Smoking |  |  |  |  |  |  |  |  |  |  |  |
|  |  | Alcohol |  |  |  |  |  |  |  |  |  |  |  |
|  |  | Length of activity |  |  |  |  |  |  |  |  |  |  |  |
| Place-related | Land- use | Shopping services |  |  |  |  |  |  |  |  |  |  |  |
|  |  | service proximity |  |  |  |  |  |  |  |  |  |  |  |
|  |  | public facility |  |  |  |  |  |  |  |  |  |  |  |
|  |  | mix-use |  |  |  |  |  |  |  |  |  |  |  |
|  |  | Facilities management |  |  |  |  |  |  |  |  |  |  |  |
|  |  | sports recreation facility |  |  |  |  |  |  |  |  |  |  |  |
|  |  | Connectivity |  |  |  |  |  |  |  |  |  |  |  |
|  |  | Accessibility services |  |  |  |  |  |  |  |  |  |  |  |
|  | Access | Traffic condition |  |  |  |  |  |  |  |  |  |  |  |
|  |  | Pavement condition |  |  |  |  |  |  |  |  |  |  |  |
|  |  | Walkable Environment |  |  |  |  |  |  |  |  |  |  |  |
|  |  | Mobility |  |  |  |  |  |  |  |  |  |  |  |
|  |  | Transportation |  |  |  |  |  |  |  |  |  |  |  |
|  | Physical form | Up keeping |  |  |  |  |  |  |  |  |  |  |  |
|  |  | Abandon buildings |  |  |  |  |  |  |  |  |  |  |  |
|  |  | Presence amenities/infrastructure sufficiency |  |  |  |  |  |  |  |  |  |  |  |
|  |  | Urban Block size |  |  |  |  |  |  |  |  |  |  |  |
|  |  | Safety |  |  |  |  |  |  |  |  |  |  |  |
|  |  | Security |  |  |  |  |  |  |  |  |  |  |  |
|  |  | green space |  |  |  |  |  |  |  |  |  |  |  |
|  | Cityscape/City Image | perceived distance |  |  |  |  |  |  |  |  |  |  |  |
|  |  | legibility |  |  |  |  |  |  |  |  |  |  |  |
|  |  | Perceived Aesthetics |  |  |  |  |  |  |  |  |  |  |  |
|  |  | Natural scenery |  |  |  |  |  |  |  |  |  |  |  |
|  | Public open spaces | Street lighting |  |  |  |  |  |  |  |  |  |  |  |
|  |  | Area open spaces ratio |  |  |  |  |  |  |  |  |  |  |  |
|  |  | Recreation Public open spaces |  |  |  |  |  |  |  |  |  |  |  |
|  |  | Quietness |  |  |  |  |  |  |  |  |  |  |  |
|  |  | cleanness |  |  |  |  |  |  |  |  |  |  |  |
|  |  | maintenance |  |  |  |  |  |  |  |  |  |  |  |
|  |  | Pollution |  |  |  |  |  |  |  |  |  |  |  |
|  |  | Landscaping quality |  |  |  |  |  |  |  |  |  |  |  |
|  | Housing | Universal design |  |  |  |  |  |  |  |  |  |  |  |
|  |  | Residential density |  |  |  |  |  |  |  |  |  |  |  |
|  |  | Residential Care Facility |  |  |  |  |  |  |  |  |  |  |  |
|  |  | Type of housing |  |  |  |  |  |  |  |  |  |  |  |
| Socio-economic environment | Social Environment | Life expectancy |  |  |  |  |  |  |  |  |  |  |  |
|  |  | Quality life |  |  |  |  |  |  |  |  |  |  |  |
|  |  | Social interaction/ community activities |  |  |  |  |  |  |  |  |  |  |  |
|  |  | Happiness |  |  |  |  |  |  |  |  |  |  |  |
|  |  | Social inclusion |  |  |  |  |  |  |  |  |  |  |  |
|  |  | Social Inequalities |  |  |  |  |  |  |  |  |  |  |  |
|  |  | Social Demography |  |  |  |  |  |  |  |  |  |  |  |
|  |  | Social democracy |  |  |  |  |  |  |  |  |  |  |  |
|  |  | Participation |  |  |  |  |  |  |  |  |  |  |  |
|  |  | Social Support |  |  |  |  |  |  |  |  |  |  |  |
|  |  | Education learning |  |  |  |  |  |  |  |  |  |  |  |
|  |  | Social capital |  |  |  |  |  |  |  |  |  |  |  |
|  |  | Religious activity |  |  |  |  |  |  |  |  |  |  |  |
|  |  | Cultural events |  |  |  |  |  |  |  |  |  |  |  |
|  | Cultural Environment | Sense place |  |  |  |  |  |  |  |  |  |  |  |
|  | Economic Environment | healthcare services |  |  |  |  |  |  |  |  |  |  |  |
|  |  | limited income |  |  |  |  |  |  |  |  |  |  |  |
|  |  | insurance coverage |  |  |  |  |  |  |  |  |  |  |  |
|  |  | Socio-economic status |  |  |  |  |  |  |  |  |  |  |  |
|  |  | Affordable housing |  |  |  |  |  |  |  |  |  |  |  |
|  |  | Car ownership |  |  |  |  |  |  |  |  |  |  |  |
|  |  | Homeownership |  |  |  |  |  |  |  |  |  |  |  |
|  |  | Household income |  |  |  |  |  |  |  |  |  |  |  |
|  |  | Living situation |  |  |  |  |  |  |  |  |  |  |  |
| Governmental | Good Governance | Effective collaboration |  |  |  |  |  |  |  |  |  |  |  |
|  |  | Performance orientation |  |  |  |  |  |  |  |  |  |  |  |
|  |  | governance |  |  |  |  |  |  |  |  |  |  |  |
|  |  | Equity |  |  |  |  |  |  |  |  |  |  |  |
| Health | Physical Health | Disability |  |  |  |  |  |  |  |  |  |  |  |
|  |  | Public Health |  |  |  |  |  |  |  |  |  |  |  |
|  |  | Incidence of disease |  |  |  |  |  |  |  |  |  |  |  |
|  |  | Pain feeling |  |  |  |  |  |  |  |  |  |  |  |
|  |  | Functional ability |  |  |  |  |  |  |  |  |  |  |  |
|  |  | Risk institutionalisation |  |  |  |  |  |  |  |  |  |  |  |
|  |  | Self-reported falls |  |  |  |  |  |  |  |  |  |  |  |
|  |  | Self-reported health |  |  |  |  |  |  |  |  |  |  |  |
|  |  | Physical activity |  |  |  |  |  |  |  |  |  |  |  |
|  |  | Activities daily living |  |  |  |  |  |  |  |  |  |  |  |
|  |  | Genetic factors |  |  |  |  |  |  |  |  |  |  |  |
|  |  | BMI |  |  |  |  |  |  |  |  |  |  |  |
|  |  | Sleep hygiene |  |  |  |  |  |  |  |  |  |  |  |
|  |  | Personal hygiene |  |  |  |  |  |  |  |  |  |  |  |
|  | Mental Health | Depressive |  |  |  |  |  |  |  |  |  |  |  |
|  |  | Cognitive functioning |  |  |  |  |  |  |  |  |  |  |  |
|  |  | Psychological distress |  |  |  |  |  |  |  |  |  |  |  |
|  |  | Psychological well-being |  |  |  |  |  |  |  |  |  |  |  |
|  |  | Anxiety |  |  |  |  |  |  |  |  |  |  |  |
|  |  | Anger |  |  |  |  |  |  |  |  |  |  |  |
|  |  | Restorative activity |  |  |  |  |  |  |  |  |  |  |  |
|  |  | Spiritual activity |  |  |  |  |  |  |  |  |  |  |  |
|  |  | Self-actualisation |  |  |  |  |  |  |  |  |  |  |  |
|  | Social Health | Relation to family |  |  |  |  |  |  |  |  |  |  |  |
|  |  | Relation work |  |  |  |  |  |  |  |  |  |  |  |
|  |  | social life |  |  |  |  |  |  |  |  |  |  |  |
|  |  | sense community |  |  |  |  |  |  |  |  |  |  |  |
| 1 Please add your proposed indicators?  2 Please mention your suggestion for dimensions, principle and indicators? | | | | | | | | | | | | | |
